# “Association of Knowledge, Dietary Behaviors, and Attitudes Toward Hair Problems Among the Population in the Chattogram Division, Bangladesh: A Cross‐Sectional Study”

**DOI:** 10.1002/hsr2.73301

**Published:** 2026-09-30

**Authors:** Rakibur Rahman, Yasin Arafat, Md. Meherajul Kabir, Samiha Siddique, Tasnim Rahman, Nur e Shyma Fatema, Sakib Bin Nour, Md. Rashedur Rahman, Shuchana das, Fatema Tuz Zohura, Cinmoy Barua, Md. Masudur Rahman, Md. Sohel Rana, Mohammad Nazmul Islam

**Affiliations:** ^1^ Department of Pharmacy International Islamic University Chittagong Chittagong Bangladesh; ^2^ Department of Statistics Noakhali Science and Technology University Noakhali Bangladesh; ^3^ Department of Pharmaceutical Technology Faculty of Pharmaceutical Sciences, University of Sciences & Technology Chittagong (USTC) Chittagong Bangladesh; ^4^ Department of Pharmacy BGC Trust University Bangladesh Chittagong Bangladesh; ^5^ Department of Pharmacy Jahangirnagar University Dhaka Bangladesh

**Keywords:** Bangladesh, cross‐sectional study, dietary intake, Hair health, knowledge gap

## Abstract

**Background and Aims:**

Hair health is influenced by sociodemographic characteristics, dietary habits, and knowledge. Despite increased global attention to hair‐related concerns, research on hair care knowledge and behaviors remains scarce in low‐resource nations like Bangladesh. The purpose of this study was to determine the demographic variables associated with dietary habits, attitudes, and awareness of hair issues among residents of Bangladesh's Chattogram Division.

**Methods:**

A cross‐sectional study was conducted with 1316 individuals (66.4% female; 80.9% from urban areas) recruited by multistage random sampling. A standardized questionnaire examined demographics, hair health awareness, dietary habits (including sugar‐sweetened beverage use), and attitudes. Multinomial logistic regression and chi‐square tests were used to assess associations.

**Results:**

In spite of the fact that 78.4% of respondents identified hair loss as a concern, only 18.8% of them recognized androgenic alopecia. Females had higher knowledge levels than males (92.6% moderate/good vs 78.3%, *p* < 0.001). Living in an urban area was substantially linked to favorable attitudes (OR = 5.31, *p* = 0.001) and improved knowledge (OR = 1.67, *p* = 0.012). Living with family was associated with higher dietary consumption (OR = 1.50, *p* = 0.026). Notably, 8.7% of respondents indicated excessive use of sugar‐sweetened beverages.

**Conclusions:**

There are significant gaps in medical understanding of hair loss, especially among males and rural inhabitants. Nutrition education and culturally appropriate awareness campaigns should be among the targeted measures.

## 1 Introduction

The psychological and social effects of hair issues, such as thinning, loss, or dandruff, are substantial and affect people worldwide [1]. Hair problems are increasingly recognized as a public health issue in Bangladesh, particularly in urban areas where dietary lifestyles and environmental factors can exacerbate these conditions [2]. In this context, hair loss serves as both an aesthetic concern and an indicator of more serious medical issues, such as hormone imbalances, malnutrition, or long‐term illness [3]. Urban dwellers in Bangladesh are more likely to experience hair loss due to environmental and lifestyle factors, and the country as a whole is increasingly recognizing this as a public health issue [4]. Despite growing awareness of hair‐related concerns, there remains a lack of comprehensive information on the population's knowledge, dietary patterns, and attitudes toward hair health are evident, particularly in regions such as Chattogram Division, Bangladesh.

The impact of gender, age, and socioeconomic level on understanding and perspective on hair issues has been demonstrated in previous research [5]. Deficiencies in specific vitamins and minerals can lead to hair loss; therefore, it is essential to consume nutrient‐rich foods and limit sugar‐sweetened beverages (SSBs) to maintain healthy hair [6, 7]. Nevertheless, the majority of prior studies have concentrated on Western populations, whereas there is a dearth of data from South Asian nations, such as Bangladesh [8]. In Bangladesh, inadequate knowledge about therapies supported by research and unhealthy eating habits, including a high intake of sugar‐sweetened beverages (SSB), are contributors to hair loss [9, 10, 11]. In Bangladesh, research on the relationship between knowledge, dietary behaviors, and attitudes toward hair health remains scarce, even as hair disorders become increasingly common. Understanding the population's knowledge, attitudes, and practices regarding hair disorders is essential for developing effective public health policies. Although studies suggest that women and urban residents tend to be more knowledgeable, awareness of medical treatments remains limited [12, 13]. Cultural norms, such as the widespread use of hair oil, may also discourage individuals from seeking professional [14]. Previous research on hair care has largely overlooked the behavioral and social aspects in favor of clinical therapies and genetic variables [15]. In the context of Bangladesh, there has been limited investigation into how gender, age, and location influence hair‐related knowledge and attitudes. This gap underscores the need for comprehensive studies to better understand the factors affecting hair health in this region.

The purpose of this study is to examine the relationship between knowledge, dietary habits, and attitudes toward hair issues among people of the Chattogram Division, Bangladesh. The goals of the study are threefold: first, to assess the level of public knowledge regarding hair problems and their management; second, to evaluate the influence of dietary habits on hair health; and third, to explore how attitudes toward hair issues are associated with demographic factors. The findings aim to provide valuable insights that can guide targeted interventions and awareness campaigns to promote hair health in this community.

## 2 Materials and Methodology

### 2.1 Study Design and Setting

The purpose of this cross‐sectional study was to investigate the relationship between individuals' attitudes, dietary habits, and awareness of hair problems in the Chattogram division, Bangladesh. The study was conducted in both rural and urban areas to ensure that the sample was representative of the population. The location was selected because it offered a broad demographic and socioeconomic backdrop, which was necessary for studying people's awareness of and practices around hair health. Data collection took place from June 2023 to February 2024, with individuals recruited using a multistage random sampling strategy to enhance the generalizability of the findings.

### 2.2 Study Sample

Participants had to meet these criteria: be at least 18 years old, live in the Chattogram Division (urban or rural), and be able to provide informed consent freely. Those who were ineligible included anyone under 18, individuals with cognitive impairments that hindered their ability to consent or answer questions, people with a scalp infection, those receiving cancer chemotherapy, or individuals with unreasonable physical measurements (height ≥ 2.5 m or ≤ 1 m, weight ≥ 300 kg or ≤ 10 kg). The study focused on this geographic area by also excluding non‐residents of the Chattogram Division.

### 2.3 Sampling and Sample Size

A method of random sampling with multiple stages was employed to ensure a representative sample of the population [16, 17]. The standard formula [18] was employed to determine the sample size.

$$n=N/[1+(N\times d^{2})]$$

where *n* and *N* represent the estimated sample and population sizes of the study area, respectively. Note that the symbol d represents a level of precision or a tolerance of 5% for the standard error [19]. The sample size was determined to be 396. However, we enrolled 1316 participants to increase statistical power for multivariable analyses, improve precision for subgroup analyses (by gender, domicile, and age groups), and minimize potential non‐response (estimated at 20%). In subgroup comparisons, this larger sample size has sufficient power (≥ 80%) to detect small‐to‐moderate effect sizes. The initial step was to randomly choose districts (Chattogram, Cox's Bazar, Rangamati, Feni, and Noakhali) from the Chattogram Division. The second step involved the random selection of rural and urban regions within these districts. Ultimately, we invited one eligible participant from each household residing in these locations to participate. Among the 1316 participants in the study, the majority were female (66.4%), and a substantial percentage lived in metropolitan areas (80.9%). The sample size was determined to be sufficient to identify statistically significant connections.

### 2.4 Data Collection Tool

A closed‐ended questionnaire [20] was established to collect data on four primary areas demographics, knowledge of hair problems, dietary intake behavior, daily hair care routines, and attitudes about hair health. The demographic variables that were taken into consideration included the following: gender, age (18–20, 21–23, 24‐27, and above 27), urban/rural residence, family income, living arrangement (alone, with friends, or with family), smoking status (never smoked, former smoker, current smoker), sleep duration (less than 5 h, 5–6 h, more than 6 h), use of hair oil (yes/no), and history of chronic diseases (no chronic disease, one chronic disease, multiple chronic diseases). Age groups were categorized as 18–20, 21–23, 24–27, and > 27 years based on the educational and employment transition points usual in the Bangladeshi setting. Specifically: 18–20 years corresponds to higher secondary education/college level; 21–23 years corresponds to undergraduate university education; 24–27 years corresponds to early career/graduate studies; and > 27 years corresponds to an established career/post‐graduate era. These groupings were chosen to permit meaningful comparisons across multiple life stages relevant to health behaviors and healthcare access in Bangladesh. Knowledge was evaluated using 11 items covering hair loss causes, symptoms (hair thinning, dandruff, androgenetic alopecia), and treatments (minoxidil, topical estrogens, herbal therapies, hair transplantation). Every right response received a score of 1, and every wrong response received a score of 0 (range: 0–11). Knowledge levels were divided into three categories: Good (9–11 correct answers), Moderate (5–8 correct answers), and Poor (0–4 correct answers) [21, 22, 23, 24]. Eight items were employed to assess dietary intake by examining the frequency of sugar‐sweetened beverage (SSB) consumption and other dietary patterns. A 4‐point rating system was used to code the responses: “never” = 1, “small” = 2, “middle” = 3, and “large” = 4 (range: 8–32). Dietary intake was categorized as Poor (8–16), Moderate (17–24), or Good (25–32) based on self‐reported intake and established dietary criteria [6, 25, 26, 27]. A Likert scale was used to measure attitudes, and responses ranged from negative to favorable [22, 28, 29]. The survey inquired as to the participants’ thoughts on hair issues, their openness to treatment, and their general interest in maintaining healthy hair [30, 31]. Agree” = 1, “Neutral” = 2, and “Disagree” = 3 were the codes assigned to the responses (range: 9–27). There were three categories of attitude levels: Good (0–9), Moderate (10–18), and Poor (19–24). Twenty‐five members of the target group participated in a pilot test of the questionnaire to verify its validity, reliability, and clarity. In response to participant feedback, changes were made to improve comprehension, and understanding. The final instrument was administered in person by trained volunteers to ensure consistent data collection [20].

### 2.5 Study Procedure

The study's goals and methods were presented to participants when they were approached in their homes or communities. All participants were asked to sign an informed consent form before any data was collected. To ensure understanding, the questionnaire was conducted in Bengali, the native language of the participants. Twenty‐five to 30 min was the average duration of each interview. The study team regularly supervised the data collection process over 9 months to ensure quality and handle any difficulties swiftly. There would be no repercussions for those who wished to withdraw at any moment, and their participation would remain anonymous.

### 2.6 Data Management and Analysis

The data was entered into STATA version 15.0 for analysis [32]. All data entries were double‐checked for accuracy, and missing data were handled using complete case analysis due to the low proportion of missing replies (< 5% for all variables). Participant characteristics were summarised using descriptive statistics and frequencies with percentages for categorical data. To evaluate relationships between categorical demographic variables and outcome levels (knowledge, nutritional consumption, attitude), the Pearson chi‐square test (χ^2^) was utilized. After controlling for potential confounders such as age, gender, place of residence, living arrangement, sleep duration, family history of hair loss, hair oil use, and disease history, multinomial logistic regression was used to find predictors of knowledge, dietary intake, and attitude levels (classified as poor, moderate, and good). The Brant test was used to evaluate the proportional odds assumption for ordinal logistic regression; a violation of this assumption (*p* < 0.05 for many predictors) supported the choice of multinomial logistic regression as the more suitable analytical method. Every statistical term, including OR (odds ratio), CI (confidence interval), SD (standard deviation), IQR (interquartile range), and χ^2^ (chi‐square), was defined at first usage. All statistical tests were two‐sided and had an a priori significance level of *α* = 0.05. Odds ratios (ORs) with 95% confidence intervals (CIs) are used to present the results. Statistical significance was defined as a p‐value of less than 0.05. The study protocol outlined all primary analyses, including descriptive statistics, chi‐square tests of association, and multinomial logistic regression models, prior to data collection. The Results section explicitly states that subgroup analyses by gender and place of residence were classified as exploratory studies.

### 2.7 Ethical Considerations

This study adhered to the ethical criteria outlined in the Declaration of Helsinki. The Institutional Review Board (IRB) of the International Islamic University Chittagong accepted the research protocol [Ref no. Pharm/P&D/online‐198/15‐2]. After being fully briefed on the study's goals, methods, risks, and benefits, each participant provided signed informed consent. Participants were made fully aware of their freedom to leave at any time without repercussions. Confidentiality and data protection were ensured through anonymization methods and restricted access. Throughout the research process, ethical behavior, appropriate documentation, and data integrity were guaranteed by the study's adherence to the International Council for Harmonization's ICH E6(R3) Guideline for Good Clinical Practice.

## 3 Results

### 3.1 Demographic Characteristics

The demographic features of the participants are summarized in Table 1. The study included 1316 participants, with women comprising the majority (66.4%) and men making up the remainder (33.6%). Most participants were in the 21–23 age group (52.1%), with the 18–20 age group (25.0%). Most respondents (80.9%) lived in metropolitan areas, while 19.1% resided in rural areas. Most families had a medium‐range income (87.2%), with smaller proportions in the low range (7.1% and 5.7%, respectively). Just a small number of people lived alone (4.0%) or with friends (9.0%), whereas the majority (86.7%) lived with their families. A total of 89.4% of respondents reported never having smoked. As for sleep patterns, 55.2% of respondents reported sleeping for 5–6 h each day, 32.9% reported sleeping for more than 6 h, and 11.9% reported sleeping for less than 5 h. Most respondents (76.3%) used hair oil, and 70.1% had no history of chronic disorders. 22.6% had one chronic ailment, and 7.2% had more than one. These results shed light on important variables that may influence knowledge, attitudes, and behaviors regarding hair, providing a comprehensive overview of the study population's demographic profile.

**Table 1:** Socio‐demographic characteristics.

Variables	Categories	Frequency	Percentage
Gender	Male	442	33.6
	Female	874	66.4
Age	18–20	329	25.0
	21–23	686	52.1
	24–27	160	12.2
	27<	141	10.7
Place of residence	Rural	252	19.1
	Urban	1064	80.9
Family income	Low	94	7.1
	Medium	1147	87.2
	High	75	5.7
Living arrangement	Living alone	53	4.0
	Living with friend	119	9.0
	Living with family	1144	86.9
Smoking status	Never smoker	1176	89.4
	Former smoker	67	5.1
	Current smoker	73	5.5
Daily sleep time	Less than 5 h	156	11.9
	5–6 h daily	727	55.2
	More than 6 h	433	32.9
Would you use any hair oil?	No	312	23.7
	Yes	1004	76.3
Disease history	No chronic disease	923	70.1
	One	298	22.6
	More than one	95	7.2

### 3.2 Knowledge Regarding Hair Problems

The percentage of knowledge regarding hair problems and their treatments revealed numerous significant insights (Figure 1). Androgenous alopecia was unfamiliar to 81.2% of the participants. A significant portion of respondents identified dandruff (66.6%), hair thinning (66.6%), and hair loss (78.4%) as prevalent symptoms of hair problems, whereas 63.5% recognized hair breaking as an indicator. The most widely recognized treatment options were oil (76.1%) and shampoo & soap (73.3%), with herbal remedies (52.4%) following in that sequence. Only 35.7% of people knew that minoxidil and topical estrogens were medical therapies, and even fewer knew that hair transplantation was an option.

**Figure 1:**
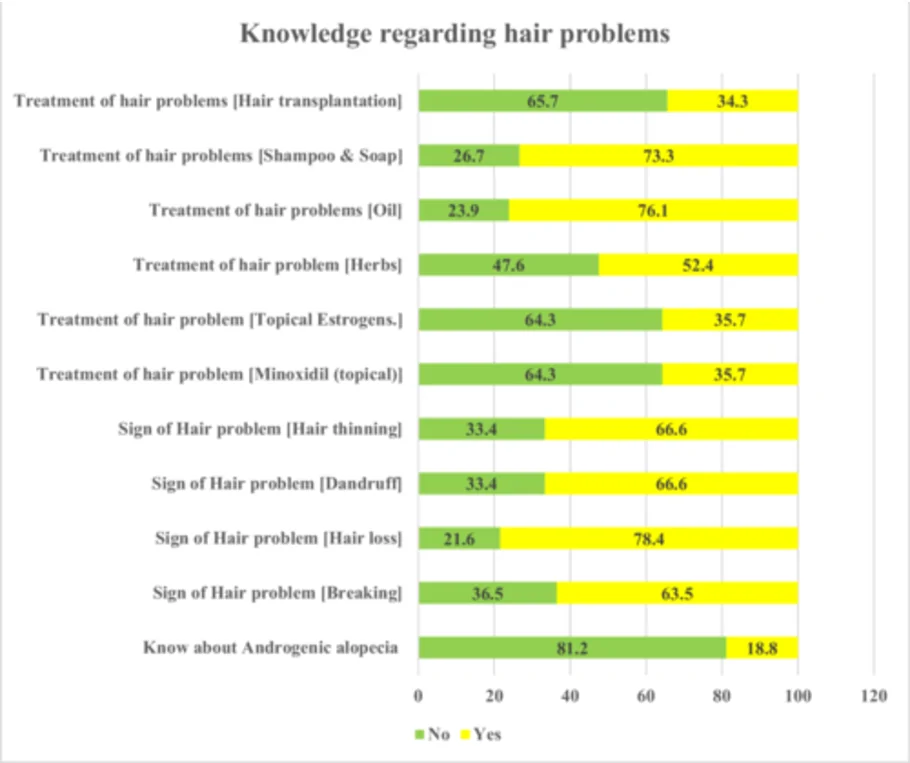
Knowledge regarding hair problems.

### 3.3 Daily Activities With Hair Problems

Figure 2 provides the most frequent reporting of the respondents' daily routines and the hair care practices. A considerable number of participants (38.1%) stated that their hair loss was unevenly distributed throughout their heads. In terms of the severity of hair loss, 46.3% said “quite a lot,” they had hair loss. Most individuals (45.4%) felt the effects of hair loss when washing their hair. The majority of respondents (64.7%) washed their hair daily, while in terms of hair washing products, the most popular choice was regular shampoo, with 56.3% of the total. 87.2% of participants reported using hair oil. As an additional point, although 59.0% of people reported doing nothing to combat hair loss. Regarding food habits, 47.4% drank a little too moderately of sugar‐sweetened beverages (SSB).

**Figure 2:**
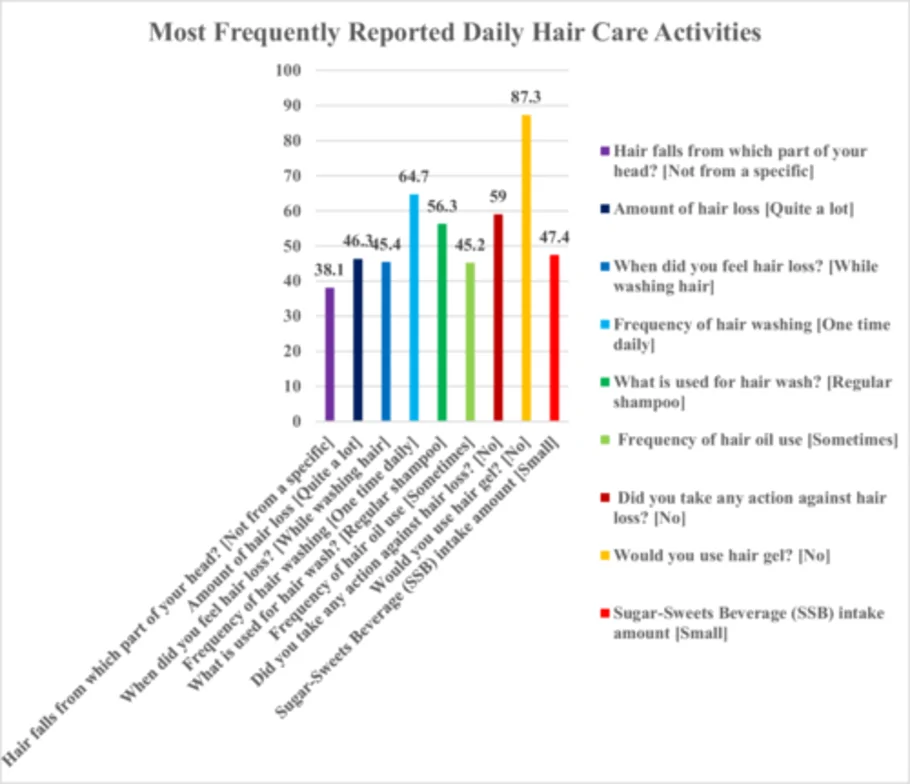
Self‐perceived hair problems and daily hair care practices among participants (*N* = 1,316).

### 3.4 Knowledge Distributed by Demographic Variable

The chi‐square test was used to analyze the relationship between demographic characteristics and knowledge levels of hair disorders, as shown in Table 2. There were notable disparities in the levels of knowledge across different demographic groups. Women showed greater levels of moderate (599) and good (210) knowledge than men, indicating a robust association between gender and knowledge (*p* < 0.001). The group of people aged 21–23 had the most significant proportion of moderate knowledge (463), and a significant correlation was found between age and this level of knowledge (*p* < 0.001). Urbanites had greater levels of moderate knowledge (704) and good knowledge (246) than their rural counterparts, suggesting that place of residence was another important predictor. Knowledge was substantially linked to a family history of hair loss.

**Table 2:** Distribution of knowledge scores across demographic variables.

Variable	Category	Knowledge			Pearson Chi‐square	*p* value
		Poor	Moderate	Good		
Gender	Male	96	266	80	56.74	0.000
	Female	65	599	210		
Age	18–20	58	217	54	24.18	0.000
	21–23	66	463	157		
	24–27	22	103	35		
	Above 27	15	82	44		
Place of Resident	Rural	47	161	44	13.61	0.001
	Urban	114	704	246		
Family Income	Low	18	52	24	7.15	0.128
	Medium	133	767	247		
	High	10	46	19		
Living Arrangement	Living alone	9	33	11	9.43	0.051
	Living with friend	17	88	14		
	Living with family	135	735	264		
Smoking Status	Never smoker	139	788	249	9.17	0.057
	Former smoker	11	34	22		
	Current smoker	11	43	19		
Sleep Time	Less than 5 h	24	82	50	17.57	0.001
	5–6 h	78	489	160		
	More than 6 h	59	294	80		
Family History of Hair Loss	No	85	265	80	34.67	0.000
	Yes	76	600	210		
Use Hair Oil	No	74	179	59	50.24	0.000
	Yes	87	686	231		
Disease History	No chronic disease	118	618	187	10.19	0.037
	One	35	192	71		
	More than one	8	55	32		

(*p* < 0.001), with individuals who reported a history of hair loss exhibiting greater levels of moderate (600) and good (210) knowledge.

The usage of hair oil was also found to be substantially associated with knowledge levels (*p* < 0.001), with users showing higher levels of knowledge than non‐users. Similarly, a statistically significant correlation was found between the amount of time people slept and their degree of knowledge. There were no significant relationships between knowledge levels and family income (*p* > 0.001), housing arrangement (*p* > 0.001), or smoking status (*p* > 0.001).

### 3.5 Dietary Intake Distributed by Demographic Variable

The chi‐square test was used to analyze the relationship between demographic characteristics and dietary intake behavior, as shown in Table 3. There were noticeable variations in the amount of food consumed across various demographic categories. The correlation between gender and food intake was statistically significant (*p* < 0.001), indicating that men had a higher percentage of healthy eating habits (135) compared to women (225). The link between age and dietary intake was statistically also significant (*p* < 0.001). The fact that people living with family reported a greater level of moderate (714) and good (311) dietary consumption compared to those living alone or with friends was not significant (*p* > 0.001). Sleep duration was not associated with greater nutritional intake (*p* > 0.001. In addition, a statistically insignificant correlation was found between disease history and food consumption (*p* > 0.001. While there were no significant relationships between food consumption behavior and household income (*p* > 0.001), smoking status (*p* > 0.001), family history of hair loss (*p* > 0.001), or hair oil use (*p* > 0.001), there was no difference in place of residence (*p* > 0.001).

**Table 3:** Distribution of dietary intake scores across demographic variables.

Variable	Category	Dietary intake			Pearson Chi‐square	*p* value
		Poor	Moderate	Good		
Gender	Male	24	283	135	17.88	0.000
	Female	111	538	225		
Age	18–20	45	197	87	20.54	0.002
	21–23	75	433	178		
	24–27	3	100	57		
	Above 27	12	91	38		
Place of resident	Rural	18	166	68	3.57	0.168
	Urban	117	655	292		
Family income	Low	10	59	25	8.15	0.086
	Medium	112	726	309		
	High	13	36	26		
Living arrangement	Living alone	5	27	21	11.32	0.023
	Living with friend	20	74	25		
	Living with family	109	714	311		
Smoking status	Never smoker	118	740	318	6.23	0.183
	Former smoker	12	37	18		
	Current smoker	5	44	24		
Sleep time	Less than 5 h	23	101	32	10.64	0.031
	5–6 h	80	447	200		
	More than 6 h	32	273	128		
Family history of hair loss	No	39	269	122	1.12	0.570
	Yes	96	552	238		
Use hair oil	No	29	191	92	1.14	0.566
	Yes	106	630	268		
Disease History	No chronic disease	111	562	250	10.77	0.029
	One	18	198	82		
	More than one	6	61	28		

### 3.6 Attitude Distributed by Demographic Variable

The chi‐square test findings demonstrated significant relationships between demographic characteristics and attitudes across multiple categories (Table 4). A highly significant correlation was found between gender and attitude (χ^2^ = 93.15, *p* < 0.001), indicating that females are more likely to hold positive attitudes than males. Urban residents reported more favorable attitudes than rural residents, and a significant connection was found between place of residence and attitude (χ^2^ = 16.86, *p* < 0.001).

**Table 4:** Distribution of attitude score across demographic variables.

Variable	Category	Attitude			Pearson Chi‐square	*p* value
		Poor	Moderate	Good		
Gender	Male	10	222	210	93.15	0.000
	Female	7	218	649		
Age	18–20	7	125	197	7.82	0.251
	21–23	7	224	455		
	24–27	2	49	109		
	Above 27	1	42	98		
Place of resident	Rural	9	96	147	16.86	0.000
	Urban	8	344	712		
Family income	Low	3	41	50	13.37	0.010
	Medium	11	376	760		
	High	3	23	49		
Living arrangement	Living alone	1	19	33	4.09	0.394
	Living with friend	1	49	69		
	Living with family	15	368	751		
Smoking status	Never smoker	15	388	773	1.03	0.906
	Former smoker	1	25	41		
	Current smoker	1	27	45		
Sleep time	Less than 5 h	1	52	103	11.75	0.019
	5–6 h	9	218	500		
	More than 6 h	7	170	256		
Family history of hair loss	No	9	167	254	12.52	0.002
	Yes	8	273	605		
Use hair oil	No	8	149	155	45.49	0.000
	Yes	9	291	704		
Disease history	No chronic disease	12	314	597	4.24	0.375
	One	5	101	192		
	More than one	0	25	70		

Furthermore, a strong correlation was found between the use of hair oil and attitude (χ^2^ = 45.49, *p* < 0.001), suggesting that individuals who use hair oil tend to hold more positive views. There were no significant associations between attitude and sleep time (χ^2^ = 11.75, *p* > 0.001), Family Income (χ^2^ = 13.37, *p* > 0.001), and a family history of hair loss (χ^2^ = 12.52,

*p* > 0.001). Nevertheless, there were no significant relationships found between attitude and characteristics such as age (χ^2^ = 7.82, *p* > 0.001), living arrangement (χ^2^ = 4.09, *p* > 0.001), smoking status (χ^2^ = 1.03, *p* > 0.001), and disease history (χ^2^ = 4.24, *p* > 0.001).

The predictors of hair health outcomes were more thoroughly examined through multivariate analysis. Variables with strong connections to the three knowledge levels (poor, moderate, and good) were identified by logistic regression. In both the Moderate and Good knowledge levels, gender was found to be a significant factor (Figure 3 and Supporting Information S1: Table S1). With odds ratios of 2.163 (*p* < 0.001) and 2.404 (*p* < 0.001), respectively, it was indicated that males were more likely to have higher knowledge levels than females. A favorable connection was also found between the place and knowledge level; odds ratios for moderate knowledge were 1.672 (*p* > 0.001) and for good knowledge were 2.140 (*p* > 0.001), indicating that city dwellers possessed more information than their rural counterparts.

**Figure 3:**
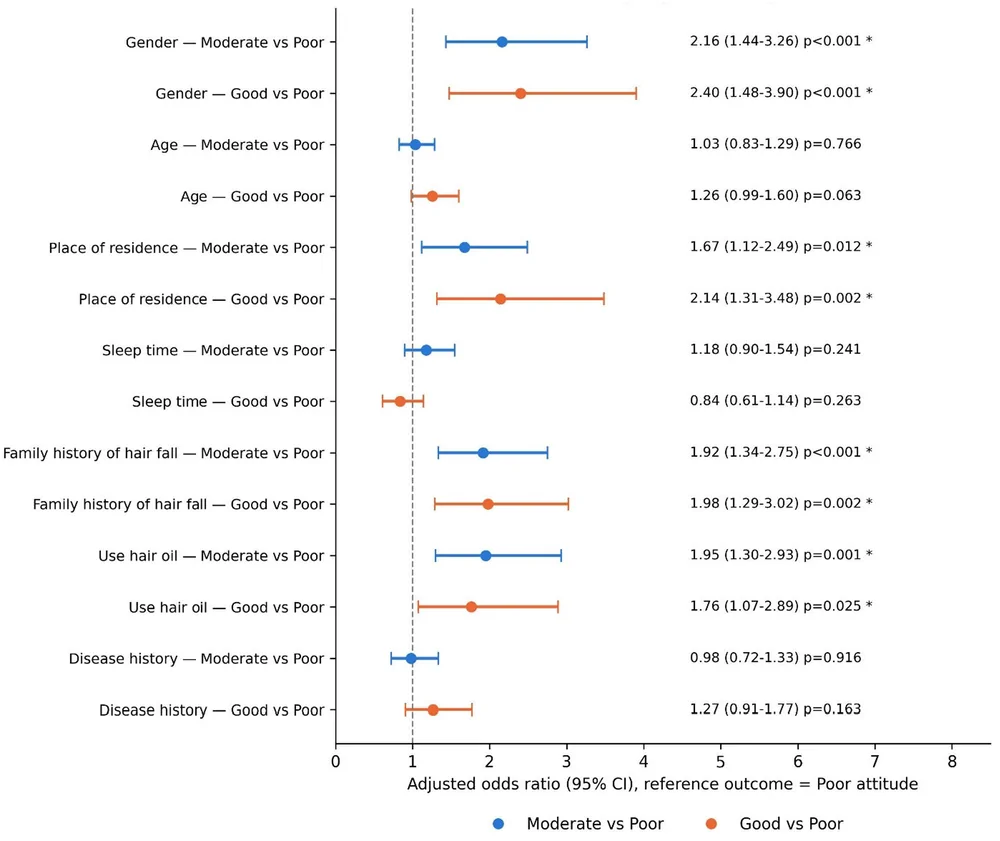
Multinomial logistic regression of knowledge.

In addition, having a family history of hair fall was found to be a significant predictor of knowledge levels, with odds ratios of 1.917 (*p* < 0.001) for Moderate knowledge and 1.977 (*p* > 0.001) for good knowledge. This suggests that people with this family history are more likely to have greater levels of knowledge. The usage of hair oil was also linked to Moderate (OR = 1.95, *p* < 0.001) and Good (OR = 1.762, *p* > 0.001) knowledge levels. Age, sleep duration, and disease history had no significant relationships with knowledge levels in this model. The significance of lifestyle and demographic factors in determining knowledge levels is underscored by these findings, which can inform the development of more targeted educational interventions (Figure 4 and Supporting Information S1: Table S2). Furthermore, the multinomial logistic regression analysis, which addressed the factors that predicted the three types of dietary intake (poor, moderate, and good), reported several significant relationships. Gender was found to be a significant predictor for both moderate and good levels of nutritional consumption. The odds ratios for these levels were 0.361 (*p* < 0.001) and 0.323 (*p* < 0.001), respectively. This suggests that females are more likely to have better dietary intake than males (Figure 4 and Supporting Information S1: Table 2). This suggests that females are more likely to have better dietary intake than males.

**Figure 4:**
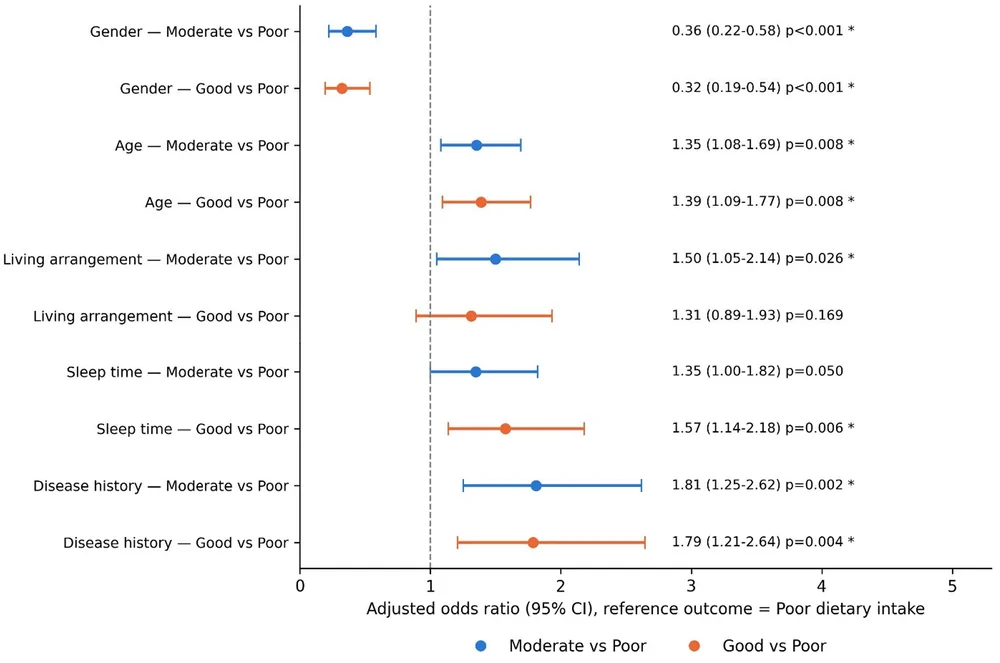
Multinomial logistic regression of dietary intake.

In this study, an illness history was found to be a strong predictor of both moderate (OR = 1.810, *p* = 0.002) and good (OR = 1.787, *p* = 0.004) food consumption levels, indicating that those with a history of disease tended to improve their eating habits (Figure 4 and Supporting Information S1: Table 2).

These results highlight the role of demographic and health‐related variables in influencing eating habits, which may guide more specific nutritional interventions. Also, the multinomial logistic regression study that investigated factors that indicated attitude levels (Poor, Moderate, and Good) showed a lot of associations, especially with the places individuals resided. The odds ratios for people residing in urban areas were 5.312 (*p* < 0.001) for good attitudes and 4.278 (*p* > 0.001) for Moderate attitudes, suggesting that people in urban areas were substantially more likely to have better attitudes than those in rural areas. There was a weak but statistically significant correlation between gender and good attitude levels (OR = 2.785, *p* > 0.001), indicating that men may generally have better attitudes than women (Figure 5 and Supporting Information S1: Table 3).

**Figure 5:**
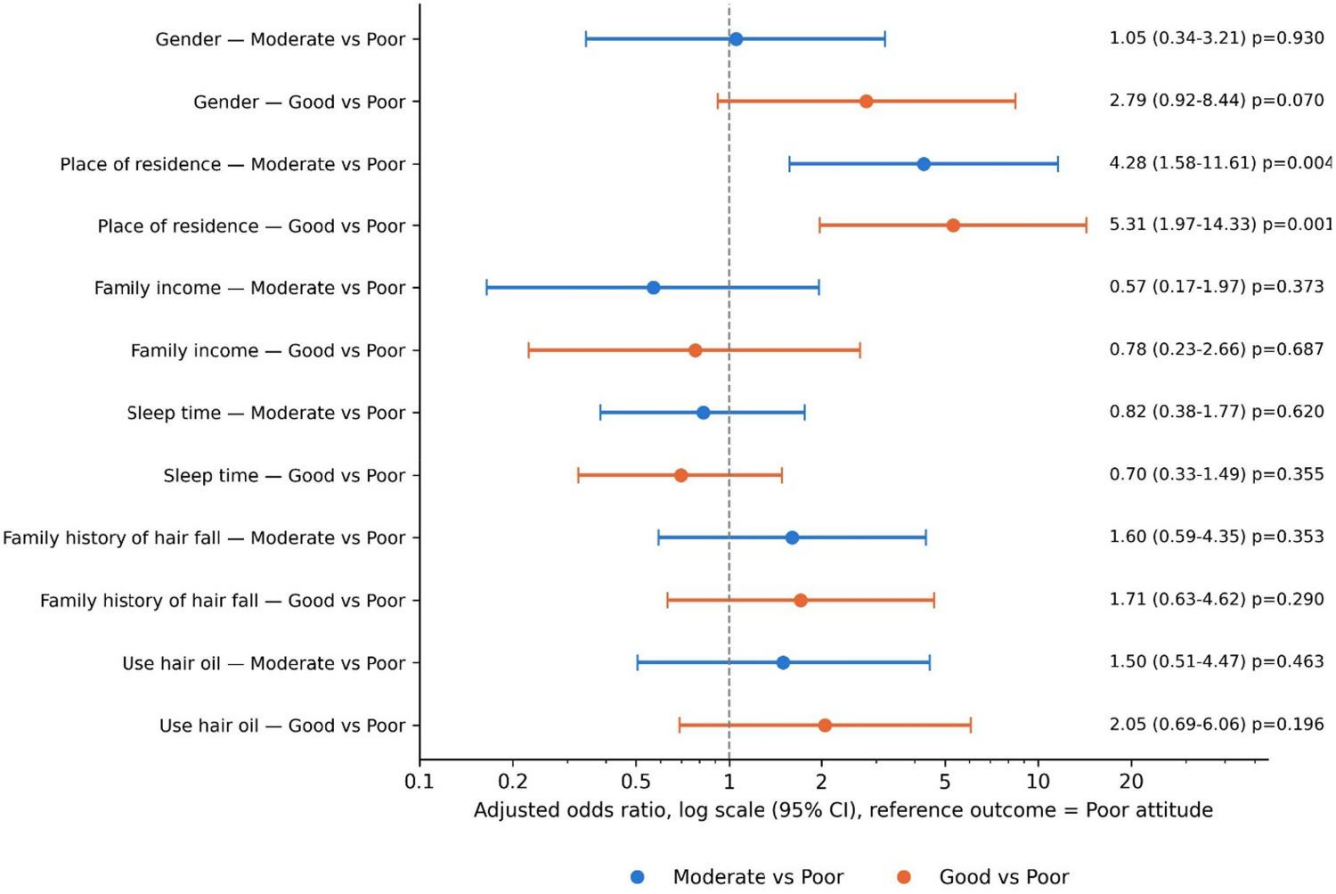
Multinomial logistic regression of attitude.

## 4 Discussion

The current study is an in‐depth analysis of the correlations between attitudes, dietary habits, and knowledge regarding hair health among 1316 participants in the Chattogram Division of Bangladesh. The results reveal notable differences in hair health awareness among demographic groups, with a notable lack of understanding regarding medical treatments for hair loss being especially prevalent. The fact that only 18.8% of participants identified androgenetic alopecia and 78.4% identified hair loss as a problem indicates that, despite the widespread recognition of observable symptoms, the understanding of underlying causes remains limited (Figure 1). These knowledge gaps were more noticeable among males and rural populations, which is consistent with global trends that show metropolitan women are generally more health‐conscious, likely due to better access to healthcare services and information [33]. The significant correlation between using hair oil and having more knowledge (moderate: 686; good: 231) emphasizes the potential of cultural practices as entry gates for health education programs (Table 2). There were notable differences in dietary patterns between the sexes and in different types of living environments, which emerged as another important factor influencing hair health. The fact that women reported a higher quality of diet (good: 225 vs. 135 for men) is likely indicative of more generalized gender disparities in health‐wise actions (Table 3). Regulated meal arrangements and the sharing of knowledge between generations may facilitate the improved eating habits of older individuals and those living with family. Given the well‐established associations between high‐sugar diets and inflammation of the hair follicles, the 8.7% of participants who reported substantial intake levels of sugar‐sweetened beverages are cause for serious worry [6]. This demographic requires specific dietary interventions, and our findings align with global research indicating that hair health is best supported by diets rich in vitamins, iron, and protein [1].

There are interesting trends in attitudes regarding hair health, particularly about gender and location. Attitudes were far more positive (good: 712) among city dwellers than among those in the countryside (good: 147), which may be attributable to variations in beauty standards and accessibility to cosmetic services. The fact that men in this sample had somewhat more positive opinions (good: 210 vs. good: 649) may be attributed to cultural standards regarding the acceptability of hair loss or reporting biases, as it runs counter to several worldwide studies (Table 4). Existing research on stress‐related hair loss is supported by the connection between sufficient sleep (more than 6h) and positive attitudes [3]. On the other hand, the cultural embeddedness of hair care practices is highlighted by the strong association with hair oil use (good: 704 users vs. 155 non‐users). These attitudes suggest that public health initiatives should employ distinct messaging strategies for urban and rural populations, as well as for men and women.

The previous study's findings are thought to be the first account of women's hair growing faster after using a nutraceutical product [34]. Several studies have shown that this dietary supplement, which contains a patented marine ingredient, can reduce hair loss, enhance hair diameter, and stimulate the growth of both thick and thin hairs since then [35, 36]. Additionally, a positive correlation was found between age and dietary quality in this study. Numerous studies have documented the adverse health effects of sugary drinks, including mortality, cardiovascular disease, obesity, and dental caries [37, 38]. Many young people still drink a lot of SSBs because they don't know any better. Potential mediators of the effect of SSB overconsumption on MPHL include long‐term health conditions and psychological issues. Among the many chronic disorders linked to SSBs, AGA is a common symptom, particularly in patients who have diabetes, hypertension, hyperglycemia, thyroid dysfunction, anemia, or hypertension [39, 40, 41, 42, 43]. The majority of people believe that hair loss is a clinical sign of these disorders. Furthermore, it is thought that SSB use can exacerbate emotional issues [44, 45]. Moderate consumption was significantly correlated with living arrangement (OR = 1.498, *p* = 0.026), while Good intake was not (OR = 1.312, *p* = 0.169). This suggests that individuals who live with family or in structured arrangements tend to eat better than those who live alone. Sleep duration had a marginally significant positive correlation with moderate dietary intake (OR = 1.349, *p* = 0.050), but a positive correlation with good dietary intake (OR = 1.574, *p* = 0.006) (Figure 5). A cohort study found that sugary foods and drinks hurt participants’ mental health over the long run [46]. A meta‐analysis found that compared to SSB nondrinkers, those who consumed three cans of cola daily had a risk of depression that was around 25% greater [47]. In a cross‐sectional study of people aged 45 and up, researchers reported that those who ate the most added simple sugars also had the highest levels of anxiety [48]. Those mental health issues could lead to Hair loss. According to a meta‐analysis and comprehensive review, a strong correlation exists between AGA and emotional states [49].

The vast majority of females suffer from severe body image issues, and global beauty standards differ significantly from one another [50]. In Bangladesh, young women are also more self‐conscious of their attractiveness [51]. This finding aligns with previous research, which has shown that women place a high value on physical appearance (72% of participants were female) [52, 53]. In this model, there were no significant relationships found between attitude levels and family income, sleep time, hair fall history in the family, or hair oil use. These results emphasize the impact of demographic and geographic factors on attitudes, which could be used to develop targeted interventions that would enhance attitudes in particular populations.

These findings can greatly improve public health studies and practices now and in the future. Education campaigns should target men and those living in rural areas, going over the basics of hair physiology and the best ways to treat it based on scientific data. To encourage hair‐healthy diets and reduce the intake of sugar‐sweetened beverages, nutrition interventions could leverage current family dynamics. There is a chance to combine conventional medical knowledge with more culturally significant practices by utilizing hair oil.

### 4.1 Limitation of the Study

There are some limitations to be aware of. First, the cross‐sectional design precludes causal inference; the links shown are correlations, not causal relationships. Second, all data were self‐reported, which could introduce recall bias (particularly for dietary intake and the timing of hair loss) and social desirability bias (especially for attitudes and health behaviors). Third, the study sample was primarily urban (80.9%) and young (52.1% aged 21–23), which might limit generalizability to elderly persons and rural populations. Fourth, and most importantly, the assessment of hair loss location, amount, severity, and timing was totally reliant on participant self‐report, with no clinical validation from objective measurements such as trichoscopy, standardized hair‐pull testing, phototrichograms, or dermatologist evaluation. These subjective measurements may be significantly influenced by recollection bias (especially regarding the timing and course of hair loss), perception bias (in which individuals may exaggerate or underestimate the severity of their hair loss), and a lack of diagnostic confirmation. As a result, the accuracy of findings about actual hair loss severity, distribution patterns, or clinical hair health status is inevitably limited. These variables should be considered as markers of participants’ perceptions of their hair health, not as objective clinical assessments. Fifth, the study's results might not apply to other parts of Bangladesh or other nations because it was geographically restricted to the Chattogram Division. Sixth, despite the large sample size, the greater number of female and urban participants may have introduced selection bias. Seventh, stress levels, medication use, hormonal status, and genetic predisposition were not evaluated as potential confounders that could potentially impact hair health.

Despite the study's limitations, it lays the groundwork for future longitudinal investigations that incorporate clinical assessments of hair health. It would be beneficial for future studies to explore the cultural influences on hair care attitudes and the biological mechanisms that link this population's diet to hair quality. Public health initiatives in Bangladesh and other similar countries can do a better job of promoting hair health as part of overall wellness if they address the information gaps and change people's habits.

## 5 Conclusion

This study provides important insights into awareness and practices related to hair health among Bangladeshi adults, revealing substantial knowledge gaps regarding medical conditions such as androgenetic alopecia. Notable demographic differences were observed, with women demonstrating higher levels of knowledge and urban residents exhibiting greater comprehension. Dietary habits were found to be significant, particularly the alarmingly high consumption of sugar‐sweetened beverages and their potential effects on hair health. While cultural practices such as hair oil use were positively associated with awareness, the study identified key areas requiring intervention, especially in rural communities and among men. A major area for improvement is the study's cross‐sectional design, which inhibits causal inferences, heightened by the possibility of recall bias in the self‐reported data. It is essential to emphasize that the hair loss aspects described in this study are not scientifically verified diagnoses but rather participants’ subjective opinions. The results of this study highlight the critical need for culturally competent public health policies and targeted educational programs to increase hair health awareness in Bangladesh and other low‐resource countries. Future longitudinal studies that include objective clinical measures of hair health, such as tracheoscopy, standardized hair‐pull testing, and dermatological evaluation, are urgently required to validate self‐reported findings, establish causal links, and assess intervention success.

## Author Contributions

**Rakibur Rahman:** conceptualization, methodology, software, data curation, formal analysis, investigation, validation, visualization, writing – original draft, writing – review and editing, resources. **Yasin Arafat:** conceptualization, methodology, software, investigation, data curation, writing – original draft. **Md. Meherajul Kabir:** conceptualization, methodology, software, data curation, investigation, validation. **Samiha Siddique:** conceptualization, methodology, software, data curation, investigation, validation. **Tasnim Rahman:** conceptualization, methodology, software, data curation, investigation, validation. **Nur e Shyma Fatema:** conceptualization, methodology, software, data curation, investigation. **Sakib Bin Nour:** conceptualization, methodology, software, data curation, investigation. **Md. Rashedur Rahman:** conceptualization, methodology, formal analysis, investigation, software, data curation, resources, writing – original draft. **Shuchana das:** conceptualization, methodology, software, data curation. **Fatema Tuz Zohura:** conceptualization, methodology, software, data curation, investigation. **Cinmoy Barua:** conceptualization, methodology, software, data curation, investigation. **Md. Masudur Rahman:** formal analysis, validation, writing – review and editing, supervision, resources, project administration. **Md. Sohel Rana:** validation, formal analysis, supervision, resources, writing – review and editing. **Mohammad Nazmul Islam:** conceptualization, investigation, formal analysis, supervision, funding acquisition, project administration, writing – original draft, writing – review and editing.

## Ethics Statement

The study was conducted according to the guidelines of the Ethical Review Committee of the International Islamic University Chittagong (Pharm/P&D/online‐198/15‐2).

## Consent

All participants gave their consent before participating in the interview. Some of the forms we upload are in a scanned PDF format.

## Conflicts of Interest

The authors declare no conflicts of interest.

## Transparency Statement

The corresponding author (Prof. Mohammad Nazmul Islam) affirms that this manuscript is an honest, accurate, and transparent account of the study being reported; that no important aspects of the study have been omitted; and that any discrepancies from the study as planned have been explained.

## Supporting information


Supporting File

## Data Availability

The data that support the findings of this study are available from the corresponding author upon reasonable request.
