# Supplementary material for: “Association of Knowledge, Dietary Behaviors, and Attitudes Toward Hair Problems Among the Population in the Chattogram Division, Bangladesh: A Cross‐Sectional Study”
Source: Health Sci Rep. 2026 Sep 30;9(10):e73301. doi: 10.1002/hsr2.73301 (PMC13628151; doi:10.1002/hsr2.73301)
Supplement: Supplementary file 1 [file HSR2-9-e73301-s001.docx]

**Association of Knowledge, Dietary Behaviors, and Attitudes Toward Hair Problems Among the Population in the Chattogram Division, Bangladesh: A Cross-Sectional Study**

**Rakibur Rahman^1^, Yasin Arafat^2^, Md. Meherajul Kabir^1,¶^, Samiha Siddique^1,¶^, Tasnim Rahman^1,¶^, Nur e Shyma Fatema^1,¶^, Sakib Bin Nour^1,¶^, Md Rashedur Rahman^2^, Shuchana das^3^, Fatema Tuz Zohura^1^, Cinmoy Barua^4^, Md. Masudur Rahman^1^, Md. Sohel Rana^5^, Mohammad Nazmul Islam^1, 5*^**

^1^Department of Pharmacy, International Islamic University Chittagong, Chittagong-4318, Bangladesh

^2^Department of Statistics, Noakhali Science and Technology University, Noakhali – 3814, Bangladesh

^3^Department of Pharmaceutical Technology, Faculty of Pharmaceutical Sciences, University of Sciences & Technology Chittagong (USTC), Bangladesh

^4^Department of Pharmacy, BGC Trust University Bangladesh, Chittagong 4381, Bangladesh

^5^Department of Pharmacy, Jahangirnagar University, Savar, Dhaka-1342, Bangladesh

^†^These authors have equally contributed.

**Corresponding Authors**

**Mohammad Nazmul Islam**, Assistant Professor, Department of Pharmacy, International Islamic University Chittagong, Chittagong-4318, Bangladesh. Email: [nazmul@iiuc.ac.bd](mailto:nazmul@iiuc.ac.bd)

**Supplementary Tables**

**Supplementary Table 1: Multinomial logistic regression of Knowledge**

| **Knowledge** | **Odds ratio** | **Std. error** | **P- value** | **95% Confidence interval** | |
| --- | --- | --- | --- | --- | --- |
| **Poor** | (base outcome) | | | | |
| **Moderate** |  | | | | |
| Const. | 0.823 | 0.241 | 0.505 | 0.464 | 1.460 |
| Gender | 2.163 | 0.453 | 0.000 | 1.435 | 3.263 |
| Age | 1.033 | 0.116 | 0.766 | 0.830 | 1.287 |
| Place of resident | 1.672 | 0.341 | 0.012 | 1.121 | 2.493 |
| Sleep time | 1.176 | 0.163 | 0.241 | 0.896 | 1.544 |
| Family history of hair fall | 1.917 | 0.353 | 0.000 | 1.337 | 2.750 |
| Use hair oil | 1.95 | 0.404 | 0.001 | 1.299 | 2.927 |
| Disease history | 0.983 | 0.153 | 0.916 | 0.725 | 1.334 |
| **Good** |  | | | | |
| Const. | 0.238 | 0.085 | 0.000 | 0.118 | 0.478 |
| Gender | 2.404 | 0.595 | 0.000 | 1.480 | 3.904 |
| Age | 1.257 | 0.155 | 0.063 | 0.987 | 1.601 |
| Place of resident | 2.140 | 0.533 | 0.002 | 1.314 | 3.485 |
| Sleep time | 0.837 | 0.133 | 0.263 | 0.614 | 1.142 |
| Family history of hair fall | 1.977 | 0.429 | 0.002 | 1.292 | 3.024 |
| Use hair oil | 1.762 | 0.445 | 0.025 | 1.075 | 2.890 |
| Disease history | 1.268 | 0.216 | 0.163 | 0.908 | 1.772 |

**Supplementary Table 2: Multinomial logistic regression of Dietary Intake**

| **Dietary Intake** | **Odds ratio** | **Std. error** | **P- value** | **95% Confidence interval** | |
| --- | --- | --- | --- | --- | --- |
| **Poor** | (base outcome) | | | | |
| **Moderate** |  | | | | |
| Const. | 2.765 | 1.178 | 0.017 | 1.200 | 6.373 |
| Gender | 0.361 | 0.089 | 0.000 | 0.223 | 0.584 |
| Age | 1.353 | 0.155 | 0.008 | 1.081 | 1.694 |
| Living Arrangement | 1.498 | 0.273 | 0.026 | 1.049 | 2.140 |
| Sleep time | 1.349 | 0.206 | 0.050 | 1.000 | 1.820 |
| Disease history | 1.810 | 0.341 | 0.002 | 1.251 | 2.619 |
| **Good** |  | | | | |
| Const. | 1.337 | 0.618 | 0.530 | 0.541 | 3.307 |
| Gender | 0.323 | 0.084 | 0.000 | 0.194 | 0.536 |
| Age | 1.389 | 0.171 | 0.008 | 1.091 | 1.769 |
| Living Arrangement | 1.312 | 0.260 | 0.169 | 0.891 | 1.933 |
| Sleep time | 1.574 | 0.261 | 0.006 | 1.137 | 2.180 |
| Disease history | 1.787 | 0.357 | 0.004 | 1.208 | 2.643 |

**Supplementary Table 3: Multinomial logistic regression of Attitude**

| **Attitude** | **Odds ratio** | **Std. error** | **P- value** | **95% Confidence interval** | |
| --- | --- | --- | --- | --- | --- |
| **Poor** | (base outcome) | | | | |
| **Moderate** |  | | | | |
| Const. | 13.580 | 12.647 | 0.005 | 2.188 | 84.259 |
| Gender | 1.052 | 0.598 | 0.930 | 0.345 | 3.208 |
| Place of resident | 4.278 | 2.178 | 0.004 | 1.577 | 11.606 |
| Family Income | 0.569 | 0.360 | 0.373 | 0.165 | 1.967 |
| Sleep time | 0.824 | 0.321 | 0.620 | 0.384 | 1.770 |
| Family history of hair fall | 1.605 | 0.817 | 0.353 | 0.592 | 4.352 |
| Use hair oil | 1.503 | 0.835 | 0.463 | 0.506 | 4.467 |
| **Good** |  | | | | |
| Const. | 8.289 | 7.702 | 0.023 | 1.341 | 51.222 |
| Gender | 2.785 | 1.575 | 0.070 | 0.919 | 8.441 |
| Place of resident | 5.312 | 2.690 | 0.001 | 1.968 | 14.334 |
| Family Income | 0.776 | 0.488 | 0.687 | 0.226 | 2.659 |
| Sleep time | 0.698 | 0.271 | 0.355 | 0.326 | 1.494 |
| Family history of hair fall | 1.710 | 0.867 | 0.290 | 0.633 | 4.618 |
| Use hair oil | 2.046 | 1.133 | 0.196 | 0.691 | 6.058 |
